# Cost-Effectiveness of HPV vaccination; Need for Economic and Social Policy Intervention

**DOI:** 10.34172/aim.2022.56

**Published:** 2022-05-01

**Authors:** Mohammad Fakhrolmobasheri, Leila Mousavi Seresht

**Affiliations:** ^1^Heart Failure Research Center, Isfahan Cardiovascular Research Institute, Isfahan University of Medical Sciences, Isfahan, Iran; ^2^Department of Obstetrics and Gynecology, Isfahan University of Medical Sciences, Isfahan, Iran

 Cervical (*cervix uteri*) cancer is a relatively common malignancy all around the globe, holding the 9^th^ place in the list of cancers sorted according to deaths per year in 2020.^1^ High-risk human papilloma viruses (HR-HPV) are the known etiologic agents contributing to the development of cervical precancerous lesion. The role of HPV, as a viral agent, in the development of cervical cancer introduces promising insights toward preventive strategies. However, it could also complicate the situation as behavioral and socioeconomic factors could affect the prevalence and epidemiologic features of the disease.^2^

 The incidence of cervical cancer varies across different regions. Sub-Saharan Africa bears the greatest burden of the disease and Asia claims the second rank of disease burden with an annual incidence of 11.7 per 100 000 female population.^3^ Based on the World Health Organization (WHO) statistics about the burden of cervical cancer and HPV infection in Iran in 2018, about 30.8 million women are at risk for cervical cancer and the annual incidence of the disease is reported to be 917 invasive cervical cancers, of which 684 cases were below the age of 65 years. Moreover, the prevalence of HR-HPV infection in women with normal cervical cytology was reported to range from 0.6% to 38.8%. It is notable that these results are derived from a limited number of studies (n = 7) published in the years 2008 to 2012 compiling the data from 3281 females.^4^ Recently in a study by Sabetet al^5^ the prevalence of HR-HPV infection in females with normal cervical cytology was reported up to 48% in a northeast Iranian population. Compared to the previous studies, this considerably high prevalence may be explained by factors influencing the rate of positive results for HR-HPV testing; firstly, most of the studies accumulate their result based on the HR-HPV test of referral patients, but not according to random population testing which might lead to falsely high estimation. Secondly, the marked variation of socioeconomic factors affecting the epidemiologic features of HR-HPV infection may either vary regionally in Iran or have undergone transformation during the time (e.g. cultural and behavioral changes in the young population).

 As WHO stated the worldwide target up to 2030, great global efforts were made to eliminate the burden of cervical cancer. The WHO global strategy for cervical cancer elimination consists of three parts including vaccination, cancer screening and treatment of precancerous and invasive cancerous lesions.^6^ In this regard, some countries sought to put the general HR-HPV vaccination for the young female population into the practice and some countries developed their national screening strategies to discover the HR-HPV infection before developing the cancerous lesions and/or the pre-malignant cervical lesion. Additionally, treatment strategies for cancerous and precancerous lesions are implemented worldwide to prevent mortality and morbidities due to cervical cancer.^7^ It should be noted that according to global cost-effectiveness studies, HPV vaccination is among the most cost-effective non-communicable disease interventions recommended by the WHO.^8^ However, in case of countries with limited resources and moderate to low burden of cervical cancer, debates would rise whether the general vaccination in the female population or general screening for HR-HPV infection would be cost-effective or not.^8,9^

**Figure 1:**
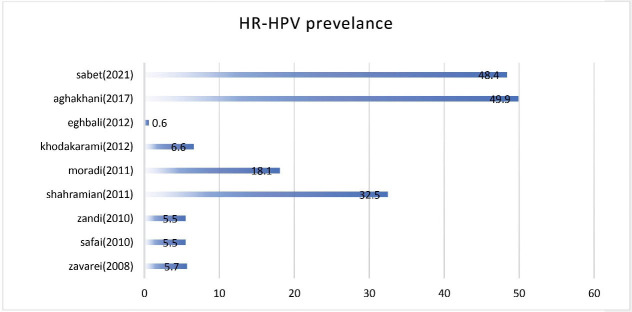
Reported Prevalence of High risk-HPV (HR-HPV; %) Infection in Iran in Females with Normal Cervical Cytology. Data source: Zavarei et al,^14^ Safai et al,^15^ Zandi et al,^16^ Sharamian et al,^17^ Moradi et al,^18^ Khodakarami et al,^13^ Eghbali et al,^19^ Aghakhani et al,^11^ Sabet et al.^5^

 In Iran, a study by Yaghoubiet al^10^ assessed the cost-effectiveness of HPV vaccination for young females (9 to 15 y/o). The authors postulated that regarding the relatively low burden of disease and relatively high costs for vaccine, it would not be cost-effective to vaccinate young girls. Although the study was professionally designed at the time, due to recent changes in the regional factors affecting the epidemiology of HR-HPV infection in Iran and changes in national economic status, it might be necessary to reconsider their conclusion. Moreover, the novel locally produced HPV vaccine which is approved by the Iranian Food and Drug Organization (FDO) is now available at a much lower cost. On the other hand, the authors had only calculated the costs of primary treatment in case of cervical cancer without considering neither the costs for treating precancerous lesions nor the cost of the patient long-term surveillance and further treatment. Regarding the psychologic burden of cervical cancer, the culture of strong emotional bonds among family members and the cultural sanctity of motherhood highlights the role of psychosocial impact of cancer in the young female population. Additionally, as the diagnosis of cervical cancer could pose a huge burden of stress on the family, many subsequent social negative effects could follow, such as stress disorders in children and other family members. The conclusion about the cost-effectiveness of HPV vaccination in Iran could be also questioned as the two recent studies by Sabet et al^5^ and Aghakhani et al^11^ were not considered as indicators for the prevalence of HPV infection. The other considerable consequence of cancerous or precancerous lesions in the young female population is the negative effect on fertility as demographic studies suggest that the Iranian population may drastically grow old in the future; thus, there is strong emphasis on female reproductivity.^12^ In addition, there is an obvious need for young people to survive in order to maintain the society age balance. Another neglected indirect expense in patients undergoing treatment for cervical cancer is the consequent charge of extensive surgical procedures or chemotherapeutic agents and/or radiotherapy side effects. Notably, it should be pointed out that recent studies of the prevalence of HR-HPV infection among the Iranian population indicate an upward trend (Figure 1) although this idea is not supported by strong evidence.

 The other concept affecting the discussion about the costs of HR-HPV infection versus vaccination is the burden for anogenital warts and other HPV-induced neoplasms, like anogenital or oropharyngeal cancer in both genders.^4^ There are also novel insights on the relation between breast cancer, as the most common malignancy in Iran, and HR-HPV infection that could even draw more attention to HPV vaccination.^13^ Another evidence in support of this commentary is a recent study by Bonjour et al^8^ that was conducted to elucidate the dilemma of HR-HPV vaccination for low-middle income countries. The authors indicated that the estimated risk of lifetime cervical cancer incidence in Iranian girls born from 2005 to 2014 would be 3600 vs. 17 005 new cases, for HR-HPV vaccination vs. no vaccination in young girls, respectively. These reduced numbers consider the optimum preventive effects of HR-HPV vaccines. Additionally, the authors predicted that Pakistan, a neighboring country of Iran with great cultural and population interaction with the Iranian population, would bear an enormous burden for HR-HPV and cervical cancer in near future. This subject should be a matter of concern for the Iranian healthcare system as the prevalence of HR-HPV in neighboring countries could also affect the epidemiology of HR-HPV infection and cervical cancer in Iran.

**Figure 2:**
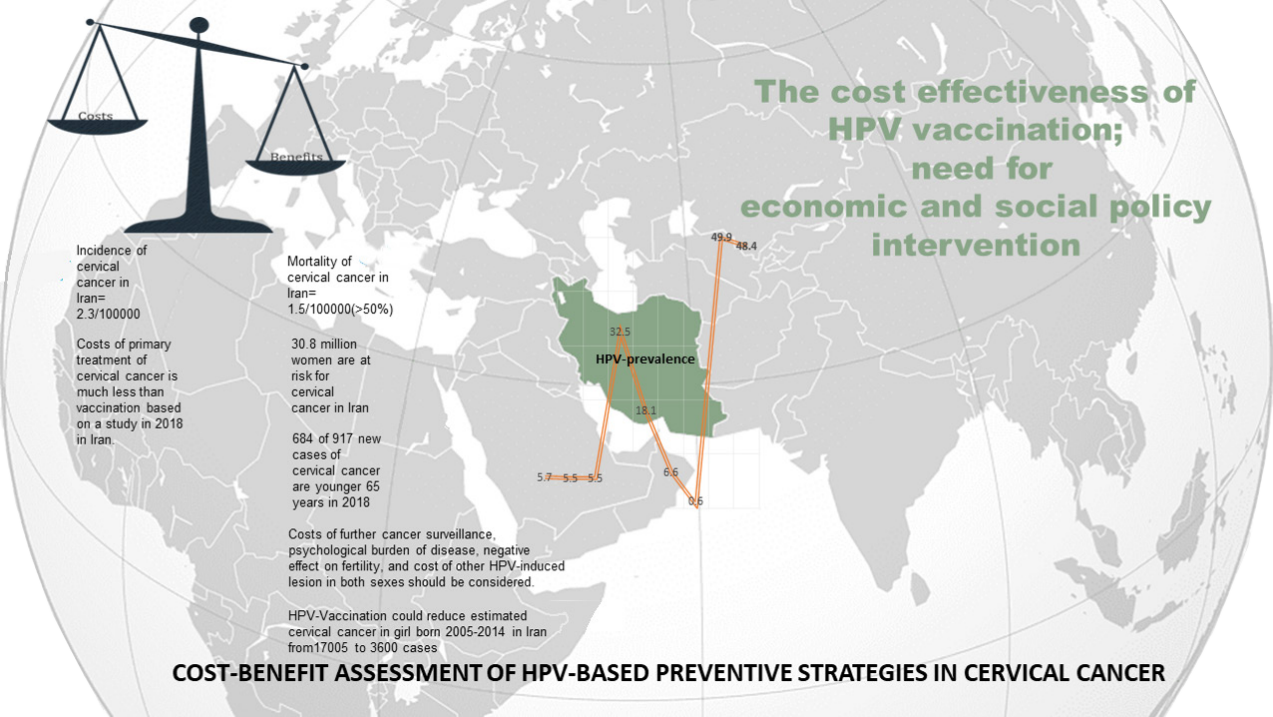
Cost-Benefit Assessment of HPV-Based Preventive Strategies in Cervical Cancer.

 Taken together, although many factors are considered in the cost-effectiveness studies about HR-HPV vaccination and testing in the general population (Figure 2), some regional and cultural factors, that may change with time, might have indirect effects not only on the target population but also on other social and economic aspects of the countries, especially as the cultural trends in developing countries favor greater social, economic and cultural roles for the female population. Hence, we suggest that reconsidering the cost-effectiveness of HR-HPV vaccination particularly with locally produced vaccines and HR-HPV testing in Iran might be profitable for both human resources and stakeholders as it could prevent a great invisible burden of psychosocial and economic problems.
